# Supplementary material for: Hazard source detection of longitudinal tearing of conveyor belt based on deep learning
Source: PLoS One. 2023 Apr 6;18(4):e0283878. doi: 10.1371/journal.pone.0283878 (PMC10079013; doi:10.1371/journal.pone.0283878)
Supplement: Attachment — Submitted filename: Response to Reviewers.docx [file pone.0283878.s001.docx]

**Original Manuscript ID:** PONE-D-22-30806

**Original Article Title:** “Hazard source detection of longitudinal tearing of conveyor belt based on deep learning”

**To:** PLOS ONE Editor

**Re:** Response to reviewers

Dear Editor,

Thank you for allowing a resubmission of our manuscript, with an opportunity to address the reviewers’ comments.

We are uploading (a) our point-by-point response to the comments (below) (Response to Reviewers), (b) an updated manuscript with yellow highlighting indicating changes (Revised Manuscript with Track Changes), and (c) a clean updated manuscript without highlights (*Manuscript).*

Best regards,

Yimin Wang et al.

**Reviewer#1, Concern # 1:**

**Author response:** The abstract must be re-written focusing on the technical aspects of the proposed model, the main experimental results, and the metrics used in the evaluation. Briefly discuss how the proposed model is superior.

**Author action:** We updated the manuscript by modify the abstract .

**Reviewer#1, Concern # 2:**

**Author response:** The contribution of the current study must be briefly discussed as bullet points in the introduction. And motivation must also be discussed in the manuscript.

**Author action:** We updated the manuscript by the contribution. We have added the contribution of this study and other changes.

**Reviewer#1, Concern # 3:**

**Author response:** The overall organization of the manuscript is not discussed anywhere in the manuscript. Please add the same in the introduction section of the manuscript.

**Author action:** We updated the manuscript by adding introduction of the overall organization.

**Reviewer#1, Concern # 4:**

**Author response:** Introduction section must discuss the technical gaps associated with the current problem.

**Author action:**We updated the manuscript by modify the introduction section .

**Reviewer#1, Concern # 5:**

**Author response:** The literature section is missing, authors are recommended to incorporate the same for better comprehensibility of the study.

**Author action:** We updated the manuscript by modify the introduction. Add Reference 6-11 to illustrate the need for this research.

**Reviewer#1, Concern # 6:**

**Author response:** Authors may include some of the relevant studies on deep learning like [https://doi.org/10.3390/bios12060393](https://doi.org/10.3390/bios12060393) and [https://doi.org/10.3390/s22082988](https://doi.org/10.3390/s22082988).

**Author action:** Yes, we consulted these two articles and got great help. And we added them to the list of references.

**Reviewer#1, Concern # 7:**

**Author response:** Authors may provide the architecture/block diagram of the proposed model for better comprehensibility of the proposed model concerning to various aspects of the proposed model.

**Author action:** Yes, we modified this part.

**Reviewer#1, Concern # 8:**

**Author response:** More explanation of the proposed model is desired on technical grounds.

**Author action:** Yes, we modified this part.

**Reviewer#1, Concern # 9:**

**Author response:** What is the size of the input image that is considered for processing and the size of the kernels?

**Author action:** Yes, we modified this part.We updated the manuscript by update table 1.

**Reviewer#1, Concern # 10:**

**Author response:** The important details like the size of the input/tensor/kernel must be discussed, and whether authors have used Stride 1 or Stride 2 must be presented. What type of activation function is being used in the current study.

**Author action:** Yes, we modified this part.We updated the manuscript by update table 1.

**Reviewer#1, Concern # 11:**

**Author response:** For how many epochs does the proposed model executed. what is the initial learning rate and after how many epochs does the model's learning rate saturated.

**Author action:** Yes, we modified this part.We updated the manuscript by update table 2.

**Reviewer#1, Concern # 12:**

**Author response:** Authors may present the loss functions for better comprehensibility of each of the models used in the proposed model.

**Author action:** Yes, we modified this part.

**Reviewer#1, Concern # 13:**

**Author response:** Majority of the figures lack the clarity, they quality is fair but they must be explained in the text and the figures must be cited. Where is the graph for testing loss and accuracy presented in the study.

**Author action:** Yes, we modified this part.

**Reviewer#1, Concern # 14:**

**Author response:** Please discuss more on the implementation platform and the dataset details as two sub-sections in the manuscript.

**Author action:** Yes, we modified this part.We updated the manuscript by update the details of the implementation platform and the dataset details.

**Reviewer#1, Concern # 15:**

**Author response:** What are the cases assumed as TP, TN, FP, FN (confusion matrix) in the current study.

**Author action:** Yes, we modified this part. You can find them on page 9 of the updated manuscript.

**Reviewer#1, Concern # 16:**

**Author response:** Authors must provide the details of hyper parameters like training loss, testing loss, training accuracy and testing accuracy.

**Author action:** Yes, we modified this part.We updated the manuscript by update table 3.

**Reviewer#1, Concern # 17:**

**Author response:** More comparative analysis with state-of-art models is desired.

**Author action:** Yes, we modified this part.You can find them on page 11 of the updated manuscript.

**Reviewer#1, Concern # 18:**

**Author response:** By considering the current form of the conclusion section, it is hard to understand by PLOS ONE Journal readers. It should be extended with new sentences about the necessity and contributions of the study by considering the authors' opinions about the experimental results derived from some other well-known objective evaluation values if it is possible.

**Author action:** Yes, we modified this part. We updated the manuscript by modify the conclusion section.

**Reviewer#2, Concern # 1:**

**Author response:** Both the abstract and introduction are not professionally written. Some sentences in abstract should be modified to make it more attractive for readers.

**Author action:** Yes, We updated the manuscript by modify the abstract .

**Reviewer#2, Concern # 2:**

**Author response:** In Introduction section, it is difficult to understand the novelty of the presented research work. This section should be modified carefully. In addition, the main contribution should be presented in the form of bullets.

**Author action:** We updated the manuscript by the contribution. We have added the contribution of this study and other changes.

**Reviewer#2, Concern # 3:**

**Author response:** The authors should also discuss the following works, which are about deep neural networks and image classification: ‘A Two-Tier Framework Based on GoogLeNet and YOLOv3 Models for Tumor Detection in MRI’, ‘Diabetic Retinopathy Detection Using VGG-NIN a Deep Learning Architecture’, ‘Traffic accident detection and condition analysis based on social networking data’, ‘Classification of skin disease using deep learning neural networks with MobileNet V2 and LSTM’

**Author action:** Yes, we consulted these two articles and got great help. And we added them to the list of references.

**Reviewer#2, Concern # 4:**

**Author response:** Figures are blurred, it is difficult to read these figures, their quality should be improved (see fig 3)

**Author action:** Yes, we modified this part.We have updated Figure 3.

**Reviewer#2, Concern # 5:**

**Author response:** Captions of the Figures not self-explanatory. The caption of figures should be self-explanatory, and clearly explaining the figure. Extend the description of the mentioned figures to make them self-explanatory.

**Author action:** Yes, We updated the manuscript by modifying some picture’s name.

**Reviewer#2, Concern # 6:**

**Author response:** The conclusion section should be revised. In addition, the future work should be properly discussed.

**Author action:** Yes, we modified this part. We updated the manuscript by modify the conclusion section.

**Reviewer#2, Concern # 7:**

**Author response:** The whole manuscript should be thoroughly revised in order to improve its English.

**Author action:** I am very sorry for the trouble I have caused you.We have sent our manuscript to our  proofreader for revision.
